# Empathy Manipulation Impacts Music-Induced Emotions: A Psychophysiological Study on Opera

**DOI:** 10.1371/journal.pone.0030618

**Published:** 2012-01-24

**Authors:** Andrei C. Miu, Felicia Rodica Balteş

**Affiliations:** Cognitive Neuroscience Laboratory, Department of Psychology, Babes-Bolyai University, Cluj-Napoca, Cluj, Romania; University of Sydney, Australia

## Abstract

This study investigated the effects of voluntarily empathizing with a musical performer (i.e., cognitive empathy) on music-induced emotions and their underlying physiological activity. N = 56 participants watched video-clips of two operatic compositions performed in concerts, with low or high empathy instructions. Heart rate and heart rate variability, skin conductance level (SCL), and respiration rate (RR) were measured during music listening, and music-induced emotions were quantified using the Geneva Emotional Music Scale immediately after music listening. Listening to the aria with sad content in a high empathy condition facilitated the emotion of nostalgia and decreased SCL, in comparison to the low empathy condition. Listening to the song with happy content in a high empathy condition also facilitated the emotion of power and increased RR, in comparison to the low empathy condition. To our knowledge, this study offers the first experimental evidence that cognitive empathy influences emotion psychophysiology during music listening.

## Introduction

Empathy refers to the capacity to understand and respond to the affective experience of another person [1]. In addition to automatic tendencies (i.e., emotional contagion) to mimic the emotional expressions of someone else [2], empathy also involves deliberate attempts to imagine what that person is thinking and feeling [3]. This role-taking ability is known as cognitive empathy [4]. The neural mechanisms involved in cognitive empathy largely overlap with those involved in emotion processing [5], [6], [7]. However, cognitive empathy also recruits brain regions associated with executive functions, which are necessary for adaptively distinguishing the self-perspective from that of others [1], [7]. Cognitively relating to the emotional experience of another induces patterns of autonomic activity equivalent to those of personal emotional imagery [6]. Moreover, empathic accuracy may depend on the degree of physiological synchrony between the observer and the target [8]. Therefore, it is useful to explore the impact of empathy on emotions using both subjective and physiological measures [9].

In the last decade, there has been a surge of interest in music and emotions [10], [11]. Psychophysiological studies have uniquely contributed to the idea that music listeners not only perceive feelings in music, but also experience genuine emotions, which are associated with congruent physiological, behavioral, and subjective changes [12], [13], [14]. Theories from cognitive science have argued that empathy plays an important role in music-induced emotions, emphasizing either emotional contagion [11], [15], or cognitive empathy [16], [17].

Scherer and Zentner [17] explained how cognitive empathy may be one of the central routes (i.e., involving the central nervous system) by which music induces emotion. Based on the performer's emotional expressions (e.g., colors of voice, facial expressions, gestures), listeners may presume that the performer experiences emotions and, by understanding that they are related to imaginary events, they may feel safe to deliberately respond with similar (even negative) emotions. The impact of musical performance may thus be related to empathic accuracy, which depends both on the target's emotional expressivity, and the observer's empathy [18], [19]. In addition, musical experience may be like an “affective sandbox”, by allowing listeners to pursue emotion exploration and hypothesis testing in safe environments [16].

Researchers have recently started to explore these hypotheses. An experience sampling study [20] asked participants to identify the psychological mechanisms by which the music they listened to induced emotions. Together with brain stem responses, emotional contagion was the most frequently reported (i.e., 32% of all cases), significantly more often than episodic memory, visual imagery, evaluative conditioning, music expectancy, and cognitive appraisal [11]. Autonomic and facial feedback [13], as well as activation of the brain's mirror neuron system [15], [21] may underlie the involvement of emotional contagion in music-induced emotions. Using a measure of “music empathy” (i.e., a cognitive style of processing music, rather than a general trait), Garrido and Schubert [22] found that it explained a significant portion of the variance in the enjoyment of negative music. Finally, trait empathy was found to predict wonder during a live opera performance [23]. Although these results are important, they only support the association between trait empathy and music-induced emotions. Experimental studies that manipulate empathy during music listening are necessary in order to support the hypothesis that empathy is a mechanism of music-induced emotions. For instance, Juslin et al. [11] recently emphasized that “[…] it is necessary to conduct experiments in the laboratory where factors that seem important on the basis of field studies are manipulated in a systematic (albeit necessarily simplified) manner” (p. 618). Studies should also distinguish between the various facets of empathy (e.g., trait empathy, cognitive empathy).

The main aim of this study was to test the causal relationship between cognitive empathy and music-induced emotions. Cognitive empathy was manipulated during music listening and we measured music-induced emotions and physiological activity. We used two music stimuli with sad and happy content, respectively, in order to test the effects of empathy on emotions induced by music with divergent emotional valence. The general hypothesis was that, in comparison to the low empathy condition, the high empathy condition would increase music-induced emotions and physiological activity. Considering that multimodal displays of music that incorporate facial expressions, gestures and body postures in addition to sounds may facilitate empathy with the performer [16], we used video recordings of the two music pieces performed in concerts.

## Methods

### Ethics statement

The participants signed an informed consent to participate in this study and the procedures complied with the recommendations of the AMA's Declaration of Helsinki for human studies.

### Participants

N = 56 participants (47 women; mean age = 22.4 years) were included in this study. They had no significant musical education. None of the participants reported cardiovascular or neurological conditions. They were not on medication (e.g., anxiolytics, beta-adrenergic antagonists) that would have interfered with the physiological measures used in this study. The participants were asked to refrain from alcohol, caffeine and smoking at least four hours before the experiment.

### Materials

We used two musical stimuli: *Gelido in ogni vena*, an aria from the opera *Il Farnace* by Antonio Vivaldi; and *Rataplan*, an operatic song by Maria Malibran. Both were performed by Cecilia Bartoli in concerts that are available on commercial DVDs. The aria describes the pain of a mother who is about to lose her sons, whereas the song describes the happy march of a boy-drummer who accompanies a victorious military battalion. Table 1 presents the arousal and valence scores for the two stimuli, previously obtained from an independent group of N = 61 participants (50 women). These scores show that the two music stimuli were equally unfamiliar, induced the same degree of emotional arousal, but they differed on emotional valence. In the present study, both videos presented close-ups of the performer who supported her singing with facial expressions and gestures, and they were subtitled in Romanian. The recordings were presented on a 20-inch monitor, using loudspeakers. Before the start of the experiment, a test tone was played and the participants had the opportunity to adjust the loudness to an individually comfortable level.

**Table 1:** Familiarity, emotional arousal and emotional valence scores of the two music stimuli.

Music stimuli	Familiarity	Emotional arousal	Emotional valence
*Gelido*	3.5±0.19	4.05±0.09	3.05±0.15**
*Rataplan*	3.31±0.19	4.23±0.12	2.3±0.14

Note: *Numbers in cells are means ±1 standard error. Five-step Likert scales for familiarity (1, familiar to 5, unfamiliar), emotional arousal (1, calm to 5, energetic) and emotional valence (1, pleasant to 5, unpleasant) were used.*

**p<0.01.

### Self-report measures

The Positive and Negative Affect Schedule (PANAS) was used to measure mood before the experiment (i.e., in the past few weeks until present) [24]. The Toronto Empathy Questionnaire (TEQ) measures trait empathy defined as accurate affective insight into the feeling state of another [25]. The Geneva Emotional Music Scales (GEMS) was used to quantify the following music-induced emotions: wonder, transcendence, tenderness, nostalgia, peacefulness, power, joyful activation, tension, and sadness [26].

### Physiological measures

Electrocardiogram (ECG), skin conductance, and respiration were continuously recorded during the experiment, using a Biopac MP150 system and specific electrodes and transducers.

#### Heart rate and heart rate variability

ECG was recorded using disposable pregelled Ag/AgCl electrodes placed in a modified lead II configuration, at a sample rate of 500 samples per second and amplified using an ECG100C module. After visual inspection of the recordings and editing to exclude artifacts in AcqKnowledge 3.9.0.17, all the recordings were analyzed using Nevrokard 7.0.1 (Intellectual Services, Ljubljana, Slovenia). We calculated heart rate (HR), and HR variability (HRV) indices in the frequency domains: power in the high frequency (HF) band (0.15–0.4 Hz in adults), and the low frequency (LF) (0.05–0.15 Hz) band of HRV, as well as LF/HF ratios. The latter three measures, obtained by spectral analysis, are reported in normalized units [27]. HF reflects vagal modulation of the heart, whereas LF reflects a complex interplay between sympathetic and vagal influences [28], [29], [30]. These measures were derived from each baseline and experimental conditions. The statistical analyses of HF included respiration rate (RR) as covariate in order to control for the influence of respiration on this measure.

#### Skin conductance level

After cleaning and abrading the skin of the palms, TSD203 electrodermal response electrodes filled with isotonic gel were attached to the volar surfaces of the index and medius fingers. Skin conductance level (SCL) recordings were amplified using a GSR100C module. We estimated SCL by extracting the area under the curve (ìS/s) from each baseline and experimental condition, after the downdrift in the SCL waves was eliminated using the “difference” function of AcqKnowledge [31], [32].

#### Respiration rate

One channel of respiration was measured using a top respiration band placed on the chest, below the breast. The data were recorded with the RSP100C module and the TSD201 Transducer of the Biopac system. TSD201 can arbitrarily measure from slow to very fast thoracic and abdominal respiration patterns with no loss in signal amplitude, optimal linearity and minimal hystheresis. RR (in cycles per minute) was calculated breath by breath using AcqKnowledge software.

#### Data reduction

For physiological measures, we calculated difference scores by subtracting each baseline measure (i.e., the quiet sitting period immediately preceding each music listening condition) from the corresponding experimental condition measure [33], [34]. This procedure, which is common practice in experimental psychophysiology [35], [36], allowed us to control for individual or sex-related differences in physiological activity. The raw scores were transformed to T scores for normalization.

### Procedure

At the arrival to the laboratory, each participant completed the PANAS and TEQ. After a habituation period during which participants were explained that several non-invasive recordings will be taken during music listening, the physiological electrodes for SCL and ECG, as well as the respiration transducer were attached. Then, they watched each of the recordings with the instructions for low or high empathy (adapted from [37]). In the high empathy condition, participants were instructed to imagine as vividly as possible how the performer feels about what is described in the music, and try to feel those emotions themselves; in the low empathy condition, they were instructed to take an objective perspective toward what is described in the music, and try not to get caught up in how the performer might feel. The order of the musical stimuli, and the empathy conditions were counterbalanced. The physiological activity was recorded during the music listening intervals. Each condition was preceded by a 5 minutes interval during which baseline physiological recordings were made. The participants completed GEMS right after each music listening episode, being specifically requested to rate the emotions they felt while they listened to the music. Participants completed the first condition and unless they wanted a break, they moved on to the following condition. We also asked participants to identify the stimulus dimension that contributed most to the emotions that developed during the music videos: (1) music; (2) song's lyrics; or (3) interpreter's facial expressions.

### Statistical analysis

We used Student t-tests and ANOVA to check if there were significant differences between the low and high empathy groups. We compared each emotion score derived from GEMS and physiological measure between the group who watched *Gelido* in the low empathy condition and the group who watched *Gelido* in the high empathy condition. The same comparison between empathy conditions was repeated for the other music stimulus, *Rataplan*. The comparisons were corrected for multiple comparisons: *p*≤0.005 was considered as the threshold of statistical significance for the comparisons on GEMS scores; and *p*≤0.008 for the comparisons on physiological measures. Simple regression was used to determine whether trait empathy was related to music-induced emotions and physiological activity. The data are reported in the graphs as mean ± one standard error of the mean (SEM). All the analyses were run in SPSS.

## Results

### Manipulation checks

There were no significant differences in mood or dispositional empathy between the low and high empathy groups (see Table 2). The majority of the participants attributed the emotions that they developed while they watched *Gelido* (89.69%) or *Rataplan* (76.54%) to music, and only a minority attributed these emotions to the interpreter's facial expressions or the song's lyrics.

**Table 2:** Individual differences in mood and dispositional empathy.

Condition	Mood(PANAS Scores)		Dispositional Empathy(TEQ Scores)
	*Positive* *Affect*	*Negative* *Affect*	
*Low (Manipulated) Empathy*	33.46±1.03	21.92±1.18	63.85±1.14
*High (Manipulated) Empathy*	33.74±1.12	21.8±1.17	64.37±1.23

Note: *Numbers in cells are means ±1 standard error. Abbreviations: PANAS, Positive and Negative Affect Schedule; TEQ, Toronto Empathy Questionnaire.*

### Dispositional empathy

**Figure 1:**
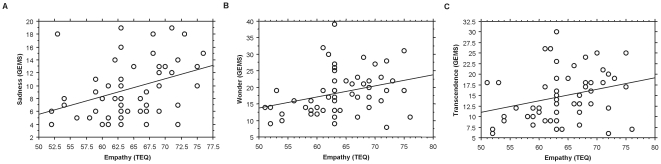
Relationships between trait empathy and music-induced sadness (A), wonder (B) and transcendence (C) after listening to *Gelido*. Abbreviations: TEQ, Toronto Empathy Questionnaire; GEMS, Geneva Emotional Music Scales.

Regression analyses indicated that the individual differences in empathy significantly predicted sadness (*R^2^* = 0.131, *β* = 0.363, *p* = 0.01), wonder (*R^2^* = 0.092, *β* = 0.303, *p* = 0.041), and transcendence (*R^2^* = 0.08, *β* = 0.282, *p* = 0.05) reported after listening to *Gelido* (see Fig. 1). Trait empathy was not significantly related to the emotions reported after listening to *Rataplan*, or to the physiological activity during either one of the arias.

### Manipulated empathy

**Figure 2:**
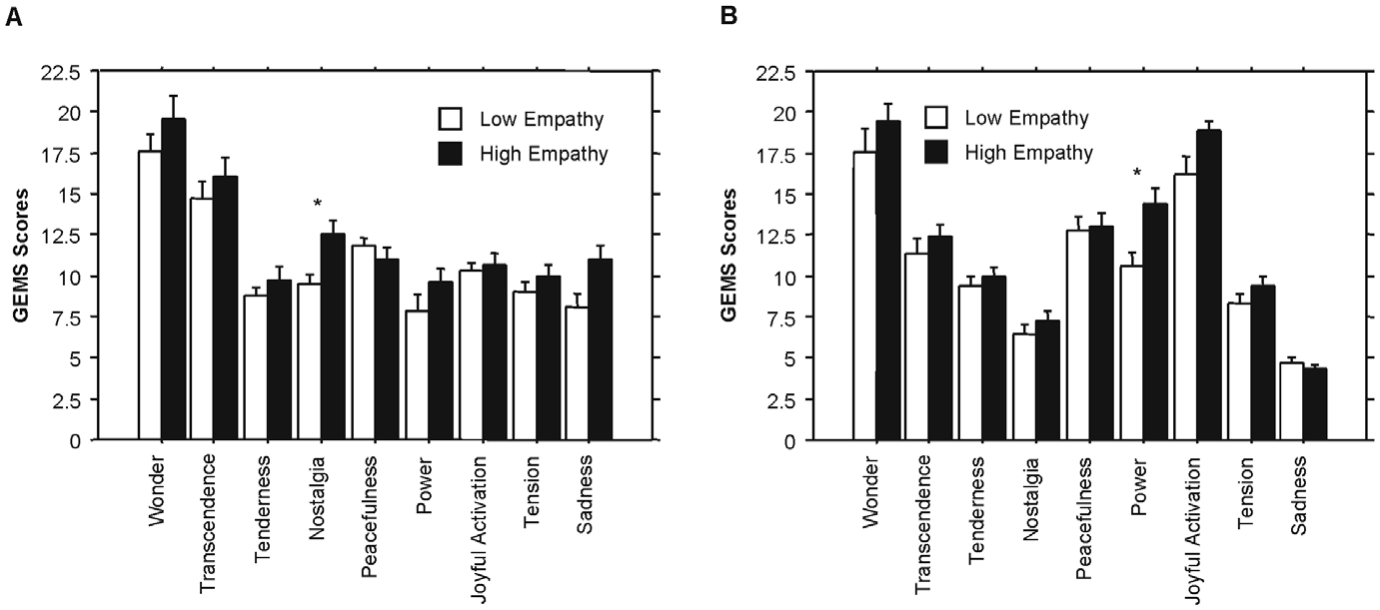
Comparison between the low and high empathy conditions on music-induced emotions after listening to *Gelido* (A) and *Rataplan* (B). Abbreviation: GEMS, Geneva Emotional Music Scales. * *p*<0.005.

Compared to the low empathy condition, the high empathy condition increased the levels of nostalgia (*F*[3, 54] = 8.87, *p* = 0.004, Cohen's *d* = 0.87) reported after listening to *Gelido* (see Fig. 2A), and the levels of power (*F*[3, 54] = 8.15, p = 0.005, Cohen's *d* = 0.84) after listening to *Rataplan* (see Fig. 2B). In order to confirm these results, we repeated the analyses on two randomly selected groups that comprised 50% of the sample each. The significant effect of empathy manipulation on nostalgia after *Gelido* was confirmed in both groups (*F*[3, 26] = 4.47, *p* = 0.005, Cohen's *d* = 0.83 in group 1; *F*[3, 26] = 4.75, *p* = 0.005, Cohen's *d* = 0.9 in group 2). Similarly, the effect of empathy manipulation on power after *Rataplan* was confirmed in both groups (*F*[3, 26] = 11.3, p = 0.000, Cohen's *d* = 1.96 in group 1; *F*[3, 26] = 6.3, *p* = 0.002, Cohen's *d* = 0.45 in group 2).

**Figure 3:**
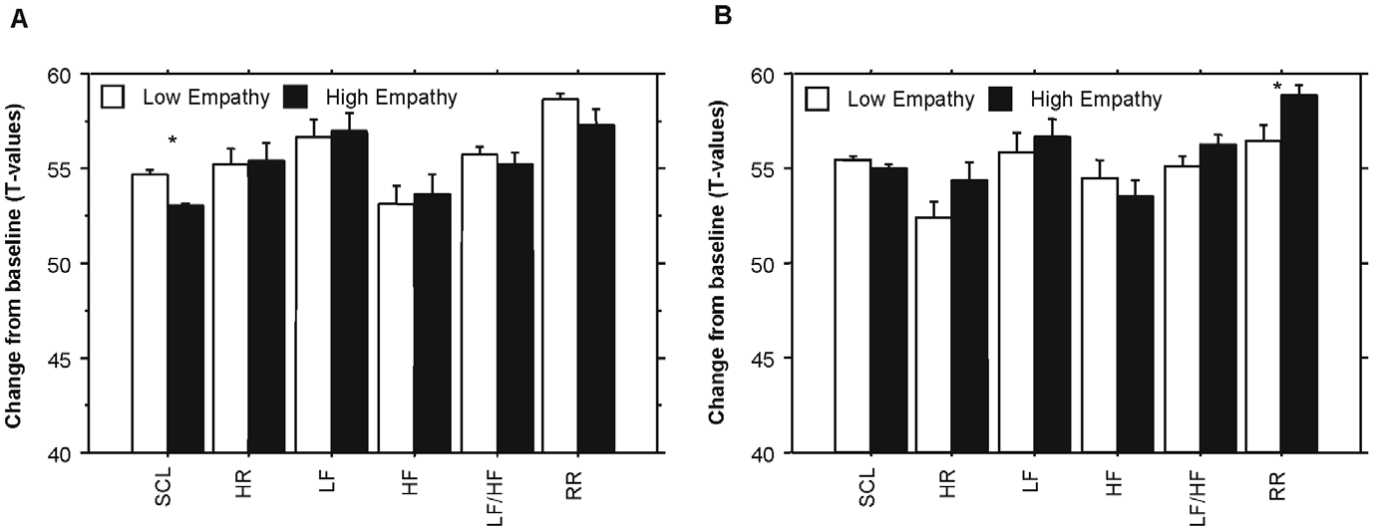
Comparison between the low and high empathy conditions on physiological activity during *Gelido* (A) and *Rataplan* (B). Abbreviations: SCL, skin conductance level, HR, heart rate; LF, power in the low frequency band of heart rate variability; HF, power in the high frequency band of heart rate variability; RR, respiration rate. * *p*<0.008.

In addition, in comparison to the low empathy condition, the high empathy manipulation decreased SCL (*F*[3, 54] = 12.06, *p* = 0.006, Cohen's *d* = 0.84) during *Gelido* (see Fig. 3A), and increased RR (*F*[3, 54] = 10.19, *p* = 0.000, Cohen's *d* = 1.03) during *Rataplan* (see Fig. 2B). All the analyses were run on two randomly selected groups, each comprising 50% of the sample. The effect of empathy manipulation on SCL during *Gelido* was confirmed in both groups (*F*[3, 26] = 4.53, *p* = 0.006, Cohen's *d* = 1.12 in group 1; *F*[3, 26] = 4.2, *p* = 0.008, Cohen's *d* = 0.7 in group 2). The effect of empathy manipulation on RR during *Rataplan* was also confirmed in both groups (*F*[3, 26] = 6.61, *p* = 0.000, Cohen's *d* = 1.24 in group 1; *F*[3, 26] = 4.3, *p* = 0.008, Cohen's *d* = 0.93 in group 2).

## Discussion

Two lines of evidence reported here strongly connect empathy and music-induced emotions. The most important was based on the manipulation of cognitive empathy and showed that the deliberate efforts made by listeners to empathize with the performer and imagine her feelings related to the music she performed, facilitated music-induced emotions and physiological activity.

These effects were highly specific in two ways. First of all, the emotions that empathy facilitated were closely related to the emotional content of the music: in the high compared to the low empathy conditions, nostalgia increased after listening to *Gelido*, whereas power increased after listening to *Rataplan*. The control analyses (run on two randomly selected groups that each comprised half of the participants) clearly confirmed these effects. This underscores the notion that empathy is based on the understanding of the target's state of mind [1], [16], which in this case referred to thoughts and feelings of the performer in relation to the music. The selectivity of these effects could not have been discovered had we not used a multidimensional music-induced emotions questionnaire such as GEMS [26]. Second, empathy changed the physiological activity in a manner that was coherent with the music-induced emotions. In comparison to the low empathy, the high empathy condition decreased SCL during *Gelido*, and increased RR during *Rataplan*. The control analyses, run on two randomly selected groups from the sample, confirmed the effects on SCL and RR. Previous studies showed that music-induced sadness is associated with SCL decreases [12], [33], [38], and happiness correlates with RR increases [14], [39]. Therefore, the present study showed that cognitive empathy selectively enhanced the emotions that were related to the content of the music, and increased the coherence of these emotions with the underlying physiological changes. To our knowledge, this is the first empirical study that causally implicates cognitive empathy in the genesis of music-induced emotions, and supports the view that this mechanisms is a central route by which music induces emotions [17].

The second line of evidence was related to dispositional empathy. In the present dataset, trait empathy significantly predicted sadness, wonder and transcendence after listening to *Gelido*. This is in line with previous findings that focused on “musical empathy” and enjoyment of negative emotions in music [22], or trait empathy and emotions such as wonder experienced during a live opera performance [23]. One may wonder why trait empathy was not also related to the positive affect induced by *Rataplan*. Various studies have described interactions of empathy and emotional valence [4], [8], and would apparently support the conjecture that trait empathy is specifically related to negative emotions. We believe that such an explanation would be artificial and premature in the present context. It is possible that negative emotions (e.g., sadness) induced in the laboratory are simply more salient than positive emotions [40]. In addition, studies have only recently started to use multidimensional scales for music-induced emotions, such as GEMS [41]. It remains to be verified whether trait empathy is selectively related to emotions of a certain valence or emotions that are specifically induced by music (e.g., wonder). Future studies might also investigate whether the effects of trait empathy and voluntarily empathizing on music-induced emotions are independent or not. One may speculate that trait empathy moderates the effects of empathizing on emotions.

Considering that we exposed the participants to multimodal displays of music performance, is it correct to attribute the emotions measured in this study to music? We believe so. Previous studies suggested that watching the performer might facilitate cognitive empathy [16]. In addition, empathic accuracy seems to depend more on visual cues only when the target communicates positive emotions [18]. Therefore, we chose to use video clips from concerts, which presented close-ups of the facial expressions and gestures of the performer during singing, because we believed that this would enhance the participants' efforts to empathize with the performer. However, we argue that GEMS scores measured after each music episode reflected music-induced emotions, for two reasons. First of all, the majority of the participants to this study attributed their emotions to music. This is in line with previous findings indicating that facial expressions and gestures of the performer and presentation of the lyrics *only facilitate* (i.e., increase or decrease them, without changing their direction or quality) the emotions that music induces [42], [43], [44]. Second, we specifically instructed participants to use GEMS in order to rate the emotions that they experienced in relation to the *music*. However, future studies might want to replicate these results on emotions induced by music listening alone, and using music from various genres for comparison.

The present results have direct implications for theories that identify empathy as a central mechanism of music-induced emotions [11], [17]. To our knowledge, this is the first experimental study that supports this view. Another contribution of this study to the psychology of music is due to its focus on opera, for there are not many studies on this complex music genre [33]. In addition, this study indicates that we can use empathy to enhance aesthetic emotions in our everyday life, while we are watching live or recorded musical performance. Musical performance offers a context in which listeners seek to resonate with the feelings of the performer in relation to the music, and it is thus important to understand how people can do that. This experimental study shows that voluntarily empathizing with a musical performer can modulate negative and positive music-induced emotions, as well as their underlying physiological activity.

## References

[1] Decety J, Jackson PL (2006). A social-neuroscience perspective on empathy.. Curr Dir Psychol Sci.

[2] Hatfield E, Rapson RL, Le YL, Decety J, Ickes W (2009). Primitive emotional contagion: Recent research.. The social neuroscience of empathy.

[3] Batson CD, Sager K, Garst E, Kang M, Rubchinsky K (1997). Is empathy-induced helping due to self-other merging?. J Pers Soc Psychol.

[4] Davis MH, Hull JG, Young RD, Warren GG (1987). Emotional reactions to dramatic film stimuli: the influence of cognitive and emotional empathy.. J Pers Soc Psychol.

[5] Carr L, Iacoboni M, Dubeau MC, Mazziotta JC, Lenzi GL (2003). Neural mechanisms of empathy in humans: a relay from neural systems for imitation to limbic areas.. Proc Natl Acad Sci U S A.

[6] Preston SD, Bechara A, Damasio H, Grabowski TJ, Stansfield RB (2007). The neural substrates of cognitive empathy.. Soc Neurosci.

[7] Ruby P, Decety J (2004). How would you feel versus how do you think she would feel? A neuroimaging study of perspective-taking with social emotions.. J Cogn Neurosci.

[8] Levenson RW, Ruef AM (1992). Empathy: a physiological substrate.. J Pers Soc Psychol.

[9] Rae Westbury H, Neumann DL (2008). Empathy-related responses to moving film stimuli depicting human and non-human animal targets in negative circumstances.. Biol Psychol.

[10] Hunter PG, Schellenberg EG, Jones MR, Fay RR, Popper AN (2010). Music and emotion.. Music perception.

[11] Juslin PN, Liljeström S, Västfjäll D, Lundqvist L-O, Juslin PN, Sloboda JA (2010). How does music evoke emotions? Exploring the underlying mechanisms.. Handbook of music and emotion: Theory, research, applications.

[12] Krumhansl CL (1997). An exploratory study of musical emotions and psychophysiology.. Can J Exp Psychol.

[13] Lundqvist L-O, Carlsson F, Hilmersson P, Juslin PN (2009). Emotional responses to music: Experience, expression, and physiology.. Psychology of Music.

[14] Nyklicek I, Thayer JF, Van Doornen LJP (1997). Cardiorespiratory differentiation of musically-induced emotions.. Journal of Psychophysiology.

[15] Overy K, Molnar-Szakacs I (2009). Being together in time: Musical experience and the mirror neuron system.. Music Perception.

[16] Livingstone SR, Thompson WF (2009). The emergence of music from the Theory of Mind.. Musicae Scientiae.

[17] Scherer KR, Zentner MR, Juslin PN, Sloboda JA (2001). Emotional effects of music: Production rules.. Music and Emotion: Theory and Research.

[18] Zaki J, Bolger N, Ochsner K (2009). Unpacking the informational bases of empathic accuracy.. Emotion.

[19] Zaki J, Bolger N, Ochsner K (2008). It takes two: the interpersonal nature of empathic accuracy.. Psychol Sci.

[20] Juslin PN, Liljestrom S, Vastfjall D, Barradas G, Silva A (2008). An experience sampling study of emotional reactions to music: listener, music, and situation.. Emotion.

[21] Molnar-Szakacs I, Overy K (2006). Music and mirror neurons: from motion to ‘e’motion.. Soc Cogn Affect Neurosci.

[22] Garrido S, Schubert E (2011). Individual differences in the enjoyment of negative emotion in music: A literature review and experiment.. Music Perception.

[23] Baltes FR, Miclea M, Miu AC (2011). A field study of emotions in a live opera performance: Individual differences in empathy, visual imagery, and mood..

[24] Watson D, Clark LA (1994). PANAS-X. Manual for the Positive and Negative Affect Schedule - Expanded form.

[25] Spreng RN, McKinnon MC, Mar RA, Levine B (2009). The Toronto Empathy Questionnaire: scale development and initial validation of a factor-analytic solution to multiple empathy measures.. J Pers Assess.

[26] Zentner M, Grandjean D, Scherer KR (2008). Emotions evoked by the sound of music: characterization, classification, and measurement.. Emotion.

[27] Task Force of the European Society of Cardiology and the North American Society of Pacing and Electrophysiology (1996). Heart rate variability: standards of measurement, physiological interpretation and clinical use.. Circulation.

[28] Eckberg DL (1997). Sympathovagal balance: a critical appraisal.. Circulation.

[29] Kingwell BA, Thompson JM, Kaye DM, McPherson GA, Jennings GL (1994). Heart rate spectral analysis, cardiac norepinephrine spillover, and muscle sympathetic nerve activity during human sympathetic nervous activation and failure.. Circulation.

[30] Miu AC, Heilman RM, Miclea M (2009). Reduced heart rate variability and vagal tone in anxiety: trait versus state, and the effects of autogenic training.. Auton Neurosci.

[31] Bechara A, Damasio H, Damasio AR, Lee GP (1999). Different contributions of the human amygdala and ventromedial prefrontal cortex to decision-making.. J Neurosci.

[32] Miu AC, Heilman RM, Houser D (2008). Anxiety impairs decision-making: psychophysiological evidence from an Iowa Gambling Task.. Biol Psychol.

[33] Baltes FR, Avram J, Miclea M, Miu AC (2011). Emotions induced by operatic music: Psychophysiological effects of music, plot, and acting A scientist's tribute to Maria Callas.. Brain Cogn.

[34] Kreibig SD, Wilhelm FH, Roth WT, Gross JJ (2007). Cardiovascular, electrodermal, and respiratory response patterns to fear- and sadness-inducing films.. Psychophysiology.

[35] Bach DR, Flandin G, Friston KJ, Dolan RJ (2009). Time-series analysis for rapid event-related skin conductance responses.. J Neurosci Methods.

[36] Wainer H (1991). Adjusting for differential base rates: Lord's paradox again.. Psychol Bull.

[37] Van Lange PA (2008). Does empathy trigger only altruistic motivation? How about selflessness or justice?. Emotion.

[38] Khalfa S, Isabelle P, Jean-Pierre B, Manon R (2002). Event-related skin conductance responses to musical emotions in humans.. Neurosci Lett.

[39] Bernardi L, Porta C, Casucci G, Balsamo R, Bernardi NF (2009). Dynamic interactions between musical, cardiovascular, and cerebral rhythms in humans.. Circulation.

[40] Rottenberg J, Ray RR, Gross JJ, Coan JA, Allen JJB (2007). Emotion elicitation using films.. The handbook of emotion elicitation and assessment.

[41] Trost W, Ethofer T, Zentner M, Vuilleumier P (2011). Mapping Aesthetic Musical Emotions in the Brain.. Cereb Cortex.

[42] Ali SO, Peynirciolu ZF (2006). Songs and emotions: are lyrics and melodies equal partners?.. Psychology of Music.

[43] Dahl S, Friberg A (2007). Visual perception of expressiveness in musicians' body movements.. Music Perception.

[44] Thompson WF, Russo FA, Quinto L (2008). Audio-visual integration of emotional cues in song.. Cognition and Emotion.

